# An empirical comparison of spatial scan statistics for outbreak detection

**DOI:** 10.1186/1476-072X-8-20

**Published:** 2009-04-16

**Authors:** Daniel B Neill

**Affiliations:** 1 HJ Heinz III College, Carnegie Mellon University, 5000 Forbes Avenue, Pittsburgh, PA 15213, USA

## Abstract

**Background:**

The spatial scan statistic is a widely used statistical method for the automatic detection of disease clusters from syndromic data. Recent work in the disease surveillance community has proposed many variants of Kulldorff's original spatial scan statistic, including expectation-based Poisson and Gaussian statistics, and incorporates a variety of time series analysis methods to obtain expected counts. We evaluate the detection performance of twelve variants of spatial scan, using synthetic outbreaks injected into four real-world public health datasets.

**Results:**

The relative performance of methods varies substantially depending on the size of the injected outbreak, the average daily count of the background data, and whether seasonal and day-of-week trends are present. The expectation-based Poisson (EBP) method achieves high performance across a wide range of datasets and outbreak sizes, making it useful in typical detection scenarios where the outbreak characteristics are not known. Kulldorff's statistic outperforms EBP for small outbreaks in datasets with high average daily counts, but has extremely poor detection power for outbreaks affecting more than  of the monitored locations. Randomization testing did not improve detection power for the four datasets considered, is computationally expensive, and can lead to high false positive rates.

**Conclusion:**

Our results suggest four main conclusions. First, spatial scan methods should be evaluated for a variety of different datasets and outbreak characteristics, since focusing only on a single scenario may give a misleading picture of which methods perform best. Second, we recommend the use of the expectation-based Poisson statistic rather than the traditional Kulldorff statistic when large outbreaks are of potential interest, or when average daily counts are low. Third, adjusting for seasonal and day-of-week trends can significantly improve performance in datasets where these trends are present. Finally, we recommend discontinuing the use of randomization testing in the spatial scan framework when sufficient historical data is available for empirical calibration of likelihood ratio scores.

## Background

Systems for automatic disease surveillance analyze electronically available public health data (such as hospital visits and medication sales) on a regular basis, with the goal of detecting emerging disease outbreaks as quickly and accurately as possible. In such systems, the choice of statistical methods can make a substantial difference in the sensitivity, specificity, and timeliness of outbreak detection. This paper focuses on methods for spatial biosurveillance (detecting clusters of disease cases that are indicative of an emerging outbreak), and provides a systematic comparison of the performance of these methods for monitoring hospital Emergency Department and over-the-counter medication sales data. The primary goal of this work is to determine which detection methods are appropriate for which data types and outbreak characteristics, with an emphasis on finding methods which are successful across a wide range of datasets and outbreaks. While this sort of analysis is essential to ensure that a deployed surveillance system can reliably detect outbreaks while keeping false positives low, most currently deployed systems which employ spatial detection methods simply use the default approaches implemented in software such as SaTScan [1].

In our typical disease surveillance task, we have daily count data aggregated at the zip code level for data privacy reasons. For each zip code *s*_*i*_, we have a time series of counts , where *t *= 0 represents the current day and *t *= 1 ... *t*_*max *_represent the counts from 1 to *t*_*max *_days ago respectively. Here we consider two types of data: hospital Emergency Department (ED) visits and sales of over-the-counter (OTC) medications. For the ED data, counts represent the number of patients reporting to the ED with a specified category of chief complaint (e.g. respiratory, fever) for that zip code on that day. For the OTC sales data, counts represent the number of units of medication sold in a particular category (e.g. cough/cold, thermometers) for that zip code on that day. Given a single data stream (such as cough and cold medication sales), our goal is to detect anomalous increases in counts that correspond to an emerging outbreak of disease. A related question, but one that we do not address here, is how to combine multiple streams of data, in order to increase detection power and to provide greater situational awareness. Recent statistical methods such as the multivariate Poisson spatial scan [2], multivariate Bayesian spatial scan [3,4], PANDA [5,6], and multivariate time series analysis [7-9] address this more difficult question, but for simplicity we focus here on the case of spatial outbreak detection using a single data stream.

For this problem, a natural choice of outbreak detection method is the *spatial scan statistic*, first presented by Kulldorff and Nagarwalla [10,11]. The spatial scan is a powerful and general method for spatial disease surveillance, and it is frequently used by the public health community for finding significant spatial clusters of disease cases. Spatial scan statistics have been used for purposes ranging from detection of bioterrorist attacks to identification of environmental risk factors. For example, they have been applied to find spatial clusters of chronic diseases such as breast cancer [12] and leukemia [13], as well as work-related hazards [14], outbreaks of West Nile virus [15] and various other types of localized health-related events.

Here we focus on the use of spatial scan methods for syndromic surveillance, monitoring patterns of health-related behaviors (such as hospital visits or medication sales) with the goal of rapidly detecting emerging outbreaks of disease. We assume that an outbreak will result in increased counts (e.g. more individuals going to the hospital or buying over-the-counter medications) in the affected region, and thus we wish to detect anomalous increases in count that may be indicative of an outbreak. Such increases could affect a single zip code, multiple zip codes, or even all zip codes in the monitored area, and we wish to achieve high detection power over the entire range of outbreak sizes. We note that this use of spatial scan statistics is somewhat different than their original use for spatial analysis of patterns of chronic illness, in which these methods were used to find localized spatial clusters of increased disease rate. One major difference is that we typically use historical data to determine the expected counts for each zip code. We then compare the observed and expected counts, in order to find spatial regions where the observed counts are significantly higher than expected, or where the ratio of observed to expected counts is significantly higher inside than outside the region.

Many recent variants of the spatial scan differ in two main criteria: the set of potential outbreak regions considered, and the statistical method used to determine which regions are most anomalous. While Kulldorff's original spatial scan approach [11] searches over circular regions, more recent methods search over other shapes including rectangles [16], ellipses [17], and various sets of irregular regions [18-20]. This paper focuses on the latter question of which statistical method to use. While Kulldorff's original approach assumes a population-based, Poisson-distributed scan statistic, recent papers have considered a variety of methods including expectation-based [21], Gaussian [22], robust [23], model-adjusted [24], and Bayesian [3,4,25] approaches.

In this study, we compare the expectation-based Poisson and expectation-based Gaussian statistics to Kulldorff's original statistic. For each of these methods, we consider four different methods of time series analysis used to forecast the expected count for each location, giving a total of 12 methods to compare. Our systematic evaluation of these methods suggests several fundamental changes to current public health practice for small-area spatial syndromic surveillance, including use of the expectation-based Poisson (EBP) statistic rather than the traditional Kulldorff statistic, and discontinuing the use of randomization testing, which is computationally expensive and did not improve detection performance for the four datasets examined in this study. Finally, since the relative performance of spatial scan methods differs substantially depending on the dataset and outbreak characteristics, an evaluation framework which considers multiple datasets and outbreak types is useful for investigating which methods are most appropriate for use in which outbreak detection scenarios.

## Methods

### The spatial scan statistic

In the spatial disease surveillance setting, we monitor a set of spatial locations *s*_*i*_, and are given an observed count (number of cases) *c*_*i *_and an expected count *b*_*i *_corresponding to each location. For example, each *s*_*i *_may represent the centroid of a zip code, the corresponding count *c*_*i *_may represent the number of Emergency Department visits with respiratory chief complaints in that zip code for some time period, and the corresponding expectation *b*_*i *_may represent the expected number of respiratory ED visits in that zip code for that time period, estimated from historical data. We then wish to detect any spatial regions *S *where the counts are significantly higher than expected.

The spatial scan statistic [11] detects clusters of increased counts by searching over a large number of spatial regions, where each region *S *consists of some subset of the locations *s*_*i*_, and finding those regions which maximize some likelihood ratio statistic. Given a set of alternative hypotheses *H*_1_(*S*) (each representing a cluster in some region *S*) and a null hypothesis *H*_0 _(representing no clusters), the likelihood ratio *F*(*S*) for a given region *S *is the ratio of the data likelihoods under the alternative and null hypotheses:



If the null or alternative hypotheses have any free parameters, we can compute the likelihood ratio statistic using the maximum likelihood parameter values [26]:



Once we have found the regions with the highest scores *F*(*S*), we must still determine which of these high-scoring regions are statistically significant, and which are likely to be due to chance. In spatial disease surveillance, the significant clusters are reported to the user as potential disease outbreaks which can then be further investigated. The regions with the highest values of the likelihood ratio statistic are those which are most likely to have been generated under the alternative hypothesis (cluster in region *S*) instead of the null hypothesis of no clusters. However, because we are maximizing the likelihood ratio over a large number of spatial regions, multiple hypothesis testing is a serious issue, and we are very likely to find many regions with high likelihood ratios even when the null hypothesis is true.

Kulldorff's original spatial scan approach [11] deals with this multiple testing issue by "randomization testing", generating a large number of replica datasets under the null hypothesis and finding the maximum region score for each replica dataset. The p-value of a region *S *is computed as , where *R*_*beat *_is the number of replica datasets with maximum region score higher than *F*(*S*), and *R *is the total number of replica datasets. In other words, a region *S *must score higher than approximately 95% of the replica datasets to be significant at *α *= .05. As discussed below, several other approaches exist for determining the statistical significance of detected regions, and these alternatives may be preferable in some cases.

### Variants of the spatial scan statistic

We consider three different variants of the spatial scan statistic: Kulldorff's original Poisson scan statistic [11] and the recently proposed expectation-based Poisson [21] and expectation-based Gaussian [22] statistics. We will refer to these statistics as KULL, EBP, and EBG respectively. Each of these statistics makes a different set of model assumptions, resulting in a different score function *F*(*S*). More precisely, they differ based on two main criteria: which distribution is used as a generative model for the count data (Poisson or Gaussian), and whether we adjust for the observed and expected counts outside the region under consideration.

The Poisson distribution is commonly used in epidemiology to model the underlying randomness of observed case counts, making the assumption that the variance is equal to the mean. If this assumption is not reasonable (i.e. counts are "overdispersed" with variance greater than the mean, or "underdispersed" with variance less than the mean), we should instead use a distribution which separately models mean and variance. One simple possibility is to assume a Gaussian distribution, and both the Poisson and Gaussian distributions lead to simple and easily computable score functions *F*(*S*). Other recently proposed spatial cluster detection methods have considered negative binomial [27], semi-parametric [28], and nonparametric [29] distributions, and these more complex model assumptions might be preferable in cases where neither Poisson nor Gaussian distributions fit the data.

A second distinction in our models is whether the score function *F*(*S*) adjusts for the observed and expected counts outside region *S*. The traditional Kulldorff scan statistic uses the ratio of observed to expected count (i.e. the observed relative risk) inside and outside region *S*, detecting regions where the risk is higher inside than outside. The expectation-based approaches (EBP and EBG) do not consider the observed and expected counts outside region *S*, but instead detect regions where the observed relative risk is higher than 1, corresponding to a higher than expected count.

All three methods assume that each observed count *c*_*i *_is drawn from a distribution with mean proportional to the product of the expected count *b*_*i *_and an unknown relative risk *q*_*i*_. For the two Poisson methods, we assume *c*_*i *_~ Poisson(*q*_*i*_*b*_*i*_), and for the expectation-based Gaussian method, we assume *c*_*i *_~ Gaussian(*q*_*i *_*b*_*i*_, *σ*_*i*_). The expectations *b*_*i *_are obtained from time series analysis of historical data for each location *s*_*i*_. For the Gaussian statistic, the variance  can also be estimated from the historical data for location *s*_*i*_, using the mean squared difference between the observed counts  and the corresponding estimated counts .

Under the null hypothesis of no clusters *H*_0_, the expectation-based statistics assume that all counts are drawn with mean *equal *to their expectations, and thus *q*_*i *_= 1 everywhere. Kulldorff's statistic assumes instead that all counts are drawn with mean *proportional *to their expectations, and thus *q*_*i *_= *q*_*all *_everywhere, for some unknown constant *q*_*all*_. The value of *q*_*all *_is estimated by maximum likelihood: , where *C*_*all *_and *B*_*all *_are the aggregate observed count ∑ *c*_*i *_and aggregate expected count ∑ *b*_*i *_for all locations *s*_*i *_respectively.

Under the alternative hypothesis *H*_1_(*S*), representing a cluster in region *S*, the expectation-based statistics assume that the expected counts inside region *S *are multiplied by some constant *q*_*in *_> 1, and thus *q*_*i *_= *q*_*in *_inside region *S *and *q*_*i *_= 1 outside region *S*. The value of *q*_*in *_is estimated by maximum likelihood. For the expectation-based Poisson statistic, the maximum likelihood value of *q*_*in *_is , where *C*_*in *_and *B*_*in *_are the aggregate count  and aggregate expectation  respectively. For the expectation-based Gaussian statistic, the maximum likelihood value of *q*_*in *_is , where  and . These sufficient statistics can be interpreted as weighted sums of the counts *c*_*i *_and expectations *b*_*i *_respectively, where the weighting of a location *s*_*i *_is inversely proportional to the coefficient of variation . The resulting likelihood ratio statistics are:



for *C*_*in *_> *B*_*in*_, and *F*_*EBP*_(*S*) = 1 otherwise.



for , and *F*_*EBG*_(*S*) = 1 otherwise. Note that these likelihood ratio statistics are only dependent on the observed and expected counts inside region *S*, since the data outside region *S *is assumed to be generated from the same distribution under the null and alternative hypotheses. Detailed derivations of these two statistics are provided in [22].

Kulldorff's scan statistic uses fundamentally different assumptions than the expectation-based statistics: under the alternative hypothesis *H*_1_(*S*) of a cluster in region *S*, it assumes that the expected counts inside and outside region *S *are multiplied by some unknown constants *q*_*in *_and *q*_*out *_respectively, where *q*_*in *_> *q*_*out*_. In this case, the maximum likelihood estimate of *q*_*in *_is  as in the expectation-based Poisson statistic, and the maximum likelihood estimate of *q*_*out *_is , where  and  respectively. The resulting likelihood ratio statistic is:



if , and *F*_*KULL*_(*S*) = 1 otherwise. Note that Kulldorff's statistic does consider the counts and expectations outside region *S*, and will only detect increased counts in a region *S *if the ratio of observed to expected count is higher inside the region than outside. Also, the term  is identical for all regions *S *for a given day of data, and can be omitted when computing the highest-scoring region. However, this term is necessary to calibrate scores between different days (e.g. when computing statistical significance). Detailed derivations of Kulldorff's statistic are found in [11] and [22].

It is an open question as to which of these three spatial scan statistics will achieve the highest detection performance in real-world outbreak detection scenarios. We hypothesize that EBG will outperform EBP for datasets which are highly overdispersed (since in this case the Poisson assumption of equal mean and variance is incorrect) and which have high average daily counts (since in this case the discrete distribution of counts may be adequately approximated by a continuous distribution). Furthermore, we note that Kulldorff's statistic will not detect a uniform, global increase in counts (e.g. if the observed counts were twice as high as expected for all monitored locations), since the ratio of risks inside and outside the region would remain unchanged. We hypothesize that this feature will harm the performance of KULL for outbreaks which affect many zip codes and thus have a large impact on the global risk . In recent work, we have shown empirically that EBP outperforms KULL for detecting large outbreaks in respiratory Emergency Department visit data [30], and we believe that this will be true for the other datasets considered here as well. However, KULL may be more robust to misestimation of global trends such as day of week and seasonality, possibly resulting in improved detection performance.

### Using the spatial scan statistic for prospective disease surveillance

In the typical prospective surveillance setting [31], we infer the expected counts for each location from the time series of historical data. Let us assume that for each spatial location *s*_*i*_, we have a time series of counts  for *t *= 0 ... *t*_*max*_, where time 0 represents the present. Our goal is to find any region *S *for which the most recent *W *counts are significantly higher than expected, where *W *denotes the temporal window size. In the more general, space-time setting, we must find the maximum of the score function *F*(*S*) over all spatial regions *S *and all temporal windows *W *= 1 ... *W*_*max*_. Space-time scan statistics are considered in detail in [21,31,32]; here we consider the purely spatial setting with a fixed window size of *W *= 1. In this simplified setting, we must compute the expected counts  for the current day from the time series of counts , using some method of time series analysis, and then find spatial regions where the current day's counts are significantly higher than expected. Here we consider four variants of the 28-day moving average (MA), with and without adjustments for day of week and seasonality. The MA method computes expectations as follows:



The moving average may be adjusted for day of week trends (MA-DOW) by computing the proportion of counts  occurring in each location *s*_*i *_on each day of the week (*j *= 1 ... 7), using 12 weeks of historical data:



When we predict the expected count for a given location on a given day, we choose the corresponding value of  and multiply our estimate by 7. This method of static adjustment for day of week assumes that weekly trends have a constant and multiplicative effect on counts for each spatial location. This is similar to the log-linear model-adjusted scan statistic proposed by Kleinman et al. [24], with the difference that we use only the most recent data rather than the entire dataset to fit the model's parameters.

The 28-day moving average takes seasonality into account by only using the most recent four weeks of data, but it may lag behind fast-moving seasonal trends, causing many false positives (if it underestimates expected counts for an increasing trend) or false negatives (if it overestimates expected counts for a decreasing trend). Thus we can perform a simple seasonal adjustment by multiplying the 28-day moving average by the ratio of the "global" 7-day and 28-day moving averages:



This "moving average with current week adjustment" (MA-WK) method has the effect of reducing the lag time of our estimates of global trends. One potential disadvantage is that our estimates of the expected counts using the 7-day average may be more affected by an outbreak (i.e. the estimates may be contaminated with outbreak cases), but using global instead of local counts reduces the variance of our estimates and also reduces the bias resulting from contamination. We can further adjust for day of week (MA-WK-DOW) by multiplying by seven times the appropriate , as discussed above.

## Results

### Datasets for evaluation

**Figure 1:**
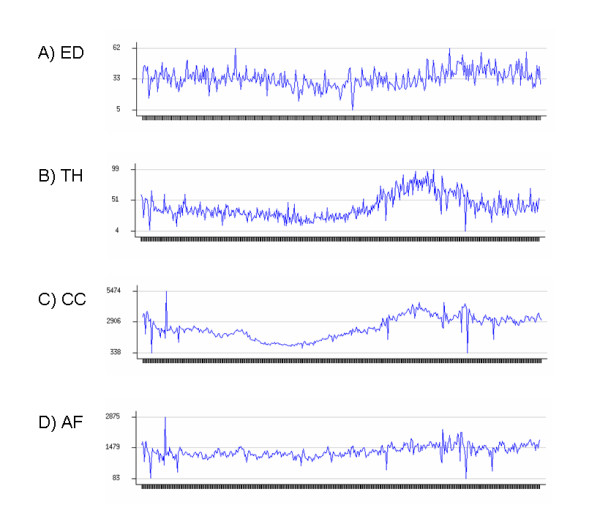
**Aggregate time series of counts for four public health datasets**. For the ED dataset, each daily count represents the total number of Emergency Department visits with respiratory chief complaints. For the three OTC datasets, each daily count represents the total number of sales of medication/medical supplies in the given product category.

We obtained four datasets consisting of real public health data for Allegheny County: respiratory Emergency Department visits from January 1, 2002 to December 31, 2002, and three categories of over-the-counter sales of medication (cough/cold and anti-fever) or medical supplies (thermometers) from October 1, 2004 to January 4, 2006. We denote these four datasets by ED, CC, AF, and TH respectively. The OTC datasets were collected by the National Retail Data Monitor [33]. Due to data privacy restrictions, daily counts were aggregated at the zip code level (by patient home zip code for the ED data, and by store zip code for the OTC data), and only the aggregate counts were made available for this study. Each count for the ED dataset represents the number of patients living in that zip code who went to an Allegheny County Emergency Department with respiratory chief complaints on that day. Each count for the OTC datasets represents the total number of sales for the given product category in monitored stores in that zip code on that day. The first 84 days of each dataset were used for baseline estimates only, giving us 281 days of count data for the ED dataset and 377 days of count data for each of the over-the-counter datasets. The ED dataset contains data for 88 distinct Allegheny County zip codes, while the three OTC datasets each contain data for 58 different Allegheny zip codes. Information about each dataset's daily counts (minimum, maximum, mean, and standard deviation) is given in Table 1, and the time series of daily counts (aggregated over all monitored zip codes) for each dataset is shown in Figure 1(a–d). We note that the CC and AF datasets have much larger average counts than the ED and TH datasets. All of the datasets exhibit overdispersion, but the OTC datasets have much more variability than the ED dataset. All four datasets demonstrate day-of-week trends, and the weekly patterns tend to vary significantly for different zip codes. Additionally, the CC and TH datasets have strong seasonal trends, while the other two datasets display much less seasonal variation. Finally, we note that all three OTC datasets have positively correlated daily counts, with coefficients of correlation *r *= .722 for the AF and CC datasets, *r *= .609 for the AF and TH datasets, and *r *= .804 for the CC and TH datasets respectively. When the counts were adjusted for known covariates (day of week, month of year, and holidays) the amount of correlation decreased, with coefficients of correlation *r *= .660 for the AF and CC datasets, *r *= .418 for the AF and TH datasets, and *r *= .462 for the CC and TH datasets respectively.

**Table 1:** Dataset description

dataset	minimum	maximum	mean	standard deviation
ED	5	62	34.40	8.34
TH	4	99	41.44	17.96
CC	338	5474	2428.46	923.47
AF	83	2875	1321.70	279.88

Minimum, maximum, mean, and standard deviation of daily counts for each of the four public health datasets (respiratory ED visits, OTC thermometer sales, OTC cough/cold medication sales, and OTC anti-fever medication sales).

### Outbreak simulations

Our first set of experiments used a semi-synthetic testing framework (injecting simulated outbreaks into the real-world datasets) to evaluate detection power. We considered a simple class of circular outbreaks with a linear increase in the expected number of cases over the duration of the outbreak. More precisely, our outbreak simulator takes four parameters: the outbreak duration *T*, the outbreak severity Δ, and the minimum and maximum number of zip codes affected, *k*_*min *_and *k*_*max*_. Then for each injected outbreak, the outbreak simulator randomly chooses the start date of the outbreak *t*_*start*_, number of zip codes affected *k*, and center zip code *s*_*center*_. The outbreak is assumed to affect *s*_*center *_and its *k *- 1 nearest neighbors, as measured by distance between the zip code centroids. On each day *t *of the outbreak, *t *= 1 ... *T*, the outbreak simulator injects Poisson(*tw*_*i *_Δ) cases into each affected zip code, where *w*_*i *_is the "weight" of each affected zip code, set proportional to its total count  for the entire dataset, and normalized so that the total weight equals 1 for each injected outbreak.

We performed three simulations of varying size for each dataset: "small" injects affecting 1 to 10 zip codes, "medium" injects affecting 10 to 20 zip codes, and "large" injects affecting all monitored zip codes in Allegheny County (88 zip codes for the ED dataset, and 58 zip codes for the three OTC datasets). For the ED and TH datasets, we used Δ = 3, Δ = 5, and Δ = 10 for small, medium, and large injects respectively. For the AF dataset, we used Δ = 30, Δ = 50, and Δ = 100, and for the CC dataset, we used Δ = 60, Δ = 100, and Δ = 200 for the three sizes of inject. We used a value of *T *= 7 for all outbreaks, and thus all outbreaks were assumed to be one week in duration. For each combination of the four datasets and the three outbreak sizes, we considered 1000 different, randomly generated outbreaks, giving a total of 12,000 outbreaks for evaluation.

We note that simulation of outbreaks is an active area of ongoing research in biosurveillance. The creation of realistic outbreak scenarios is important because of the difficulty of obtaining sufficient labeled data from real outbreaks, but is also very challenging. State-of-the-art outbreak simulations such as those of Buckeridge et al. [34], and Wallstrom et al. [35] combine disease trends observed from past outbreaks with information about the current background data into which the outbreak is being injected, as well as allowing the user to adjust parameters such as outbreak duration and severity. While the simple linear outbreak model that we use here is not a realistic model of the temporal progression of an outbreak, it is sufficient for testing purely spatial scan statistics, with the idea that we gradually ramp up the amount of increase until the outbreak is detected. The values of Δ were chosen to be large enough that most methods would eventually detect the outbreak, but small enough that we would observe significant differences in detection time between methods. It is worth noting that a large number of counts must be injected for a simulated outbreak to be detectable, especially in the CC and AF datasets. This is a common feature of syndromic surveillance methods, which rely on detecting large trends in non-specific health behaviors (as opposed to a small number of highly indicative disease findings), and limits the applicability of such methods for detecting outbreaks where only a small number of individuals are affected. Since all three methods use likelihood ratio statistics based on aggregate counts and baselines, search over the same set of regions, and do not take the shape of a region into account when computing its score, we do not expect changes in outbreak shape (e.g. circles vs. rectangles vs. irregularly shaped outbreaks) to dramatically affect the relative performance of these methods. On the other hand, variants of the spatial scan which search over different sets of regions have large performance differences depending on outbreak shape, as demonstrated in [36].

### Comparison of detection power

We tested a total of twelve methods: each combination of the three scan statistics (KULL, EBP, EBG) and the four time series analysis methods (MA, MA-DOW, MA-WK, MA-WK-DOW) discussed above. For all twelve methods, we scanned over the same predetermined set of search regions. This set of regions was formed by partitioning Allegheny County using a 16 × 16 grid, and searching over all rectangular regions on the grid with size up to 8 × 8. Each region was assumed to consist of all zip codes with centroids contained in the given rectangle. We note that this set of search regions is different than the set of inject regions used by our outbreak simulator: this is typical of real-world outbreak detection scenarios, where the size and shape of potential outbreaks are not known in advance. Additionally, we note that expected counts (and variances) were computed separately for each zip code, prior to our search over regions. As discussed above, we considered four different datasets (ED, TH, CC, and AF), and three different outbreak sizes for each dataset. For each combination of method and outbreak type (dataset and inject size), we computed the method's proportion of outbreaks detected and average number of days to detect as a function of the allowable false positive rate.

To do this, we first computed the maximum region score F* = max_*S *_*F*(*S*) for each day of the original dataset with no outbreaks injected (as noted above, the first 84 days of data are excluded, since these are used to calculate baseline estimates for our methods). Then for each injected outbreak, we computed the maximum region score for each outbreak day, and determined what proportion of the days for the original dataset have higher scores. Assuming that the original dataset contains no outbreaks, this is the proportion of false positives that we would have to accept in order to have detected the outbreak on day *t*. For a fixed false positive rate *r*, the "days to detect" for a given outbreak is computed as the first outbreak day (*t *= 1 ... 7) with proportion of false positives less than *r*. If no day of the outbreak has proportion of false positives less than *r*, the method has failed to detect that outbreak: for the purposes of our "days to detect" calculation, these are counted as 7 days to detect, but could also be penalized further.

The detection performance of each of the 12 methods is presented in Tables 2 and 3. For each combination of dataset (ED, TH, CC, AF) and outbreak size (large, medium, small), we present each method's average days to detection and percentage of outbreaks detected at a fixed false positive rate of 1/month.

**Table 2:** Comparison of detection power on ED and TH datasets, for varying outbreak sizes

method	ED large	ED medium	ED small	TH large	TH medium	TH small
KULL MA	6.05 (35.4%)	3.11 (98.4%)	3.67 (84.9%)	6.58 (12.8%)	4.36 (92.8%)	4.06 (93.1%)
KULL MA-DOW	5.74 (39.7%)	3.04 (98.3%)	3.60 (85.1%)	6.54 (12.2%)	4.36 (92.4%)	4.06 (92.2%)
KULL MA-WK	6.01 (37.0%)	3.11 (98.5%)	3.65 (85.0%)	6.57 (13.1%)	4.36 (92.8%)	4.06 (93.1%)
KULL MA-WK-DOW	5.74 (40.0%)	3.04 (98.3%)	3.61 (85.1%)	6.54 (12.3%)	4.36 (92.4%)	4.06 (92.2%)
EBP MA	**2.46 (100%)**	**2.45 (99.8%)**	**3.19 (90.1%)**	**3.31 (99.5%)**	**3.35 (99.3%)**	**3.60 (97.6%)**
EBP MA-DOW	**2.46 (100%)**	**2.44 (99.7%)**	**3.28 (90.0%)**	**3.27 (99.6%)**	**3.46 (99.3%)**	3.79 (96.5%)
EBP MA-WK	**2.50 (100%)**	**2.45 (99.7%)**	**3.18 (89.0%)**	3.53 (96.3%)	**3.39 (99.0%)**	**3.60 (96.3%)**
EBP MA-WK-DOW	**2.55 (100%)**	**2.51 (99.6%)**	**3.32 (88.5%)**	3.64 (93.2%)	3.56 (97.5%)	3.81 (94.7%)
EBG MA	2.98 (100%)	2.77 (99.9%)	3.37 (88.4%)	4.46 (88.8%)	4.34 (90.2%)	4.27 (86.6%)
EBG MA-DOW	3.04 (100%)	2.90 (99.4%)	3.41 (88.3%)	5.00 (78.4%)	4.94 (77.9%)	4.76 (74.5%)
EBG MA-WK	2.99 (99.9%)	2.78 (99.8%)	**3.35 (88.8%)**	4.71 (79.8%)	4.42 (88.2%)	4.31 (85.1%)
EBG MA-WK-DOW	3.15 (99.7%)	2.97 (98.8%)	3.40 (87.9%)	5.24 (63.2%)	5.02 (73.1%)	4.76 (73.6%)

Average days to detection, and percentage of outbreaks detected, at 1 false positive per month. Methods in bold are not significantly different (in terms of days to detect, at *α *= .05) from the best-performing method.

**Table 3:** Comparison of detection power on CC and AF datasets, for varying outbreak sizes

method	CC large	CC medium	CC small	AF large	AF medium	AF small
KULL MA	6.43 (14.8%)	2.53 (100%)	2.33 (99.5%)	6.64 (10.6%)	3.20 (100%)	3.00 (99.3%)
KULL MA-DOW	5.69 (60.8%)	**2.25 (100%)**	**2.06 (99.5%)**	6.44 (23.9%)	**2.80 (100%)**	**2.61 (99.4%)**
KULL MA-WK	6.43 (14.8%)	2.53 (100%)	2.33 (99.5%)	6.64 (10.6%)	3.20 (100%)	3.00 (99.3%)
KULL MA-WK-DOW	5.69 (60.8%)	**2.25 (100%)**	**2.06 (99.5%)**	6.44 (23.9%)	**2.80 (100%)**	**2.61 (99.4%)**
EBP MA	4.61 (87.5%)	4.07 (96.7%)	4.13 (94.0%)	**4.22 (99.4%)**	3.95 (99.9%)	3.87 (98.0%)
EBP MA-DOW	4.59 (88.6%)	4.06 (95.8%)	4.10 (94.4%)	4.70 (97.5%)	4.37 (99.0%)	4.32 (95.9%)
EBP MA-WK	3.30 (98.2%)	2.76 (100%)	2.83 (99.2%)	4.64 (82.5%)	4.07 (98.2%)	3.87 (96.7%)
EBP MA-WK-DOW	**3.07 (98.7%)**	2.58 (99.9%)	2.57 (99.3%)	4.65 (83.8%)	4.01 (98.5%)	3.89 (97.0%)
EBG MA	4.73 (80.7%)	4.30 (89.5%)	4.43 (76.5%)	4.80 (91.4%)	4.56 (94.5%)	4.47 (84.1%)
EBG MA-DOW	4.81 (80.5%)	4.36 (89.8%)	4.54 (75.0%)	4.96 (89.2%)	4.68 (93.2%)	4.70 (78.9%)
EBG MA-WK	3.73 (91.5%)	3.07 (99.4%)	3.12 (95.4%)	4.93 (75.7%)	4.47 (93.3%)	4.27 (85.9%)
EBG MA-WK-DOW	3.68 (92.4%)	3.03 (99.5%)	3.06 (96.3%)	5.04 (74.0%)	4.54 (92.0%)	4.36 (84.2%)

Average days to detection, and percentage of outbreaks detected, at 1 false positive per month. Methods in bold are not significantly different (in terms of days to detect, at *α *= .05) from the best-performing method.

For the datasets of respiratory Emergency Department visits (ED) and over-the-counter sales of thermometers (TH) in Allegheny County, the EBP methods displayed the highest performance for all three outbreak sizes, as measured by the average time until detection and proportion of outbreaks detected. There were no significant differences between the four variants of EBP, suggesting that neither day-of-week nor seasonal correction is necessary for these datasets. For small outbreaks, the EBG and KULL methods performed nearly as well as EBP (between 0.1 and 0.6 days slower). However, the differences between methods became more substantial for the medium and large outbreaks: for large outbreaks, EBG detected between 0.5 and 1.5 days slower than EBP, and KULL had very low detection power, detecting less than 40% of outbreaks and requiring over three additional days for detection.

For the dataset of cough and cold medication sales (CC) in Allegheny County, the most notable difference was that the time series methods with adjustment for seasonal trends (MA-WK) outperformed the time series methods that do not adjust for seasonality, achieving 1–2 days faster detection. The relative performance of the EBP, EBG, and KULL statistics was dependent on the size of the outbreak. However, the variants of the EBP method with adjustment for seasonality (EBP MA-WK and EBP MA-WK-DOW) were able to achieve high performance across all outbreak sizes. For small to medium-sized outbreaks, KULL outperformed EBP by a small but significant margin (0.3 to 0.5 days faster detection) when adjusted for day of week, and performed comparably to EBP without day-of-week adjustment. For large outbreaks, KULL again performed poorly, detecting three days later than EBP, and only detecting 15–61% of outbreaks (as compared to 98–99% for EBP).

For the dataset of anti-fever medication sales (AF) in Allegheny County, the results were very similar to the CC dataset, except that seasonal adjustment (MA-WK) did not improve performance. EBP methods performed best for large outbreaks and achieved consistently high performance across all outbreak sizes, while KULL outperformed EBP by about 1.2 days for small to medium-sized outbreaks. As in the other datasets, KULL had very low power to detect large outbreaks, detecting less than 25% of outbreaks and requiring more than six days to detect.

### Effects of outbreak size

**Figure 2:**
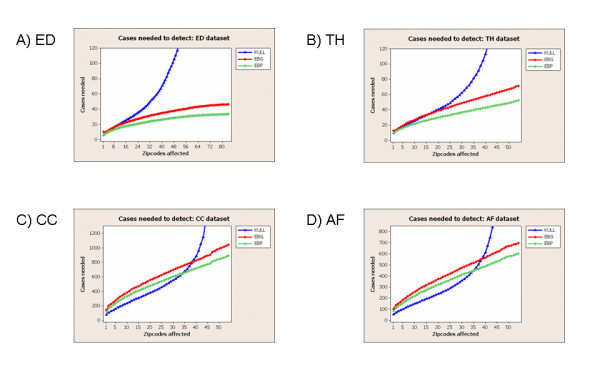
**Detectability results for four public health datasets**. Number of injected cases needed for detection of 90% of outbreaks at 1 false positive per month, as a function of outbreak size. The x-axis of each graph represents the number of Allegheny County zip codes affected by the outbreak, out of 88 monitored zip codes for the ED dataset and 58 monitored zip codes for the three OTC datasets.

To further quantify the relationship between outbreak size and detection power, we measured the average number of injected cases needed for each method to detect 90% of outbreaks at 1 false positive per month, as a function of the number of zip codes affected. For this experiment, we used the same time series method for each detection method (MA-WK for the CC dataset, and MA for the other datasets). We also used the same set of scan regions for each detection method, searching over the set of distinct circular regions centered at each zip code, as in [11]. For each combination of detection method and dataset, we computed the maximum region score F* = max_*S *_*F*(*S*) for each day of the original dataset with no outbreaks injected. We then computed the 96.7th percentile of these scores, obtaining the "threshold score" needed for detection at 1 false positive per month for that detection method for that dataset. Next we considered each distinct circular region (centered at one of the monitored zip codes) for each day of the original dataset. For each such region *S*, we recorded the number of zip codes contained in that region and computed the minimum number of injected cases (increase in count) needed for the score *F*(*S*) to become higher than the threshold. We assumed that injected cases were distributed among the zip codes in *S *proportional to the total count of each zip code. Given the number of cases needed for detection for each region, we then computed the 90th percentile of these values for each outbreak size (number of affected zip codes), giving us the average number of cases needed to detect 90% of outbreaks at the given acceptable false positive rate of 1 per month. We present these results for each of the four datasets in Figure 2(a–d).

In this experiment, we saw substantial differences in the relative performance of methods between the datasets with low average daily counts (ED and TH) and the datasets with high average daily counts (CC and AF). For the ED and TH datasets, the EBP method outperformed the EBG and KULL methods (requiring fewer injected cases for detection) across the entire range of outbreak sizes. While EBP and EBG required a number of injected cases that increased approximately linearly with the number of affected zip codes, KULL showed dramatic decreases in detection power and required substantially more injected cases when more than 1/3 of the zip codes were affected. For the CC and AF datasets, EBP and EBG again required a number of injected cases that increased approximately linearly with the number of affected zip codes, with EBP outperforming EBG. KULL outperformed EBP when less than 2/3 of the zip codes were affected, but again showed very low detection power as the outbreak size became large.

### Calibration of spatial scan statistics

In typical public health practice, randomization testing is used to evaluate the statistical significance of the clusters discovered by spatial scanning, and all regions with p-values below some threshold (typically, *α *= .05) are reported. However, randomization testing is computationally expensive, multiplying the computation time by *R *+ 1, where *R *is the number of Monte Carlo replications performed. This substantial increase in computation time, combined with the need for rapid analysis to detect outbreaks in a timely fashion, can make randomization testing undesirable or infeasible. An alternative approach is to report all regions with scores *F*(*S*) above some threshold. In this case, randomization testing is not required, but it can be difficult to choose the threshold for detection. Additionally, since the empirical distribution of scores for each day's replica datasets may be different, the regions with highest scores *F*(*S*) may not correspond exactly to the regions with lowest p-values, possibly reducing detection power.

We first examined whether the p-values produced by randomization testing are properly calibrated for our datasets. For each combination of method and dataset, we computed the p-value for each day of data with no outbreaks injected, using randomization testing with *R *= 100 replications, and reported the proportion of days that had p-values less than *α *= 0.05. For a properly calibrated method, we expect approximately 5% of the days to be significant at this level, but as can be seen from Table 4, the proportion of significant days is much higher for most methods and datasets. For the ED dataset, the two Poisson methods are properly calibrated, with false positive rates near 5%, while EBG has much higher false positive rates. For the OTC datasets, none of the methods are calibrated, with false positive rates ranging from 11–57% at *α *= .05. These results suggest that, to achieve an actual false positive rate of *α *on our datasets, we must often use a p-value threshold that is much lower than *α*. Given a sufficient amount of historical data with p-values calculated for each day, we could learn this threshold empirically, using the 100*α *percentile value from the historical data, e.g. the 5th percentile for *α *= 0.05. Alternatively, we could learn a threshold on the region score *F*(*S*) empirically, using the 100(1 - *α*) percentile of the historical data, and report all regions with scores higher than the threshold.

**Table 4:** False positive rates with randomization testing

method	ED dataset	TH dataset	CC dataset	AF dataset
KULL MA	.046	.141	.544	.358
KULL MA-DOW	.050	.146	.284	.202
KULL MA-WK	.050	.114	.568	.337
KULL MA-WK-DOW	.053	.130	.289	.186
EBP MA	.068	.162	.517	.403
EBP MA-DOW	.071	.141	.409	.340
EBP MA-WK	.064	.159	.520	.422
EBP MA-WK-DOW	.071	.149	.348	.332
EBG MA	.334	.398	.244	.204
EBG MA-DOW	.473	.496	.268	.249
EBG MA-WK	.349	.390	.218	.226
EBG MA-WK-DOW	.466	.485	.252	.249

Proportion of days significant at *α *= 0.05, for each of the four public health datasets with no outbreaks injected.

Thus we compared the detection power of each method with and without randomization testing, using empirically determined p-value and score thresholds corresponding to an actual false positive rate of *α *= .0329 (i.e. 1 false positive per month). For each combination of method and dataset, we computed the average days to detect for a set of 100 randomly generated "medium-sized" outbreaks, and compared performance using the p-value and score thresholds respectively. These results, shown in Table 5, demonstrate that randomization testing does not improve the detection power of our methods, and in most cases significantly harms performance. The poor calibration of p-values leads to many days with p-values of  in the original dataset, and since only 100 Monte Carlo replications are performed, these days cannot be reliably distinguished from the injected outbreaks.

**Table 5:** Detection power with and without randomization testing

method	ED dataset	TH dataset	CC dataset	AF dataset
KULL MA	3.23/3.23	4.41/4.23	5.40/**2.55**	4.52/**3.19**
KULL MA-DOW	3.45/3.04	4.95/**4.09**	3.73/**2.26**	3.65/**2.80**
KULL MA-WK	3.31/3.23	5.26/**4.23**	6.04/**2.55**	5.30/**3.19**
KULL MA-WK-DOW	3.19/3.04	5.20/**4.09**	3.57/**2.26**	3.99/**2.80**
EBP MA	2.54/2.50	3.95/**3.29**	6.36/**4.16**	5.89/**3.99**
EBP MA-DOW	2.65/2.53	3.51/3.44	4.59/4.10	5.62/**4.36**
EBP MA-WK	2.74/2.50	5.04/**3.40**	5.84/**2.70**	5.11/**3.92**
EBP MA-WK-DOW	2.92/2.59	4.31/**3.75**	5.05/**2.47**	5.30/**4.00**
EBG MA	4.50/**2.91**	5.90/**4.19**	4.94/4.43	4.92/4.63
EBG MA-DOW	5.48/**3.01**	5.15/4.66	5.61/**4.50**	5.00/4.79
EBG MA-WK	4.87/**2.87**	5.92/**4.24**	3.82/**3.16**	4.58/4.43
EBG MA-WK-DOW	5.53/**3.04**	5.92/**4.73**	4.90/**2.96**	4.53/4.56

Average days to detection at 1 false positive per month for "medium-sized" outbreaks injected into each dataset, using empirically determined thresholds on p-value (computed by randomization testing) and score (without randomization testing) respectively. If there is a significant difference between the detection times with and without randomization, the better-performing method is marked in bold.

One potential solution is to perform many more Monte Carlo replications, requiring a further increase in computation time. To examine this solution, we recomputed the average number of days to detection for the EBP MA method on each dataset, using *R *= 1000 Monte Carlo replications. For the ED and TH datasets, EBP MA detected outbreaks in an average of 2.45 and 3.10 days respectively; these results were not significantly different from EBP MA without randomization testing. For the CC and AF datasets, EBP MA with 1000 Monte Carlo replications detected outbreaks in 6.17 and 5.29 days respectively, as compared to 4.16 and 3.99 days for EBP MA without randomization. The significant differences in detection time for these two datasets demonstrate that, when p-values are severely miscalibrated, randomization testing harms performance even when the number of replications is large.

Recent work by Abrams et al. [37] suggests another potential solution to the miscalibration of p-values: to fit a Gumbel distribution to the distribution of maximum region scores obtained by randomization, and to compute the p-value of the discovered regions using the resulting cumulative distribution function. These "empirical/asymptotic" p-values have the benefit of being able to distinguish between very low p-values with a relatively small number of Monte Carlo replications, and thus may allow high detection power even when the p-values are miscalibrated. For example, in a severely miscalibrated dataset, without the Gumbel correction we would be unable to differentiate even extremely high scoring regions from a large proportion of the background days, since all of these regions would have an identical p-value of . With the Gumbel correction, many background days would still have very low p-values, but the extremely high scoring regions would have even lower p-values than most or all of the background days. We compare detection power of each method with and without randomization testing, using empirical/asymptotic p-values based on 100 Monte Carlo replications, in Table 6. For most combinations of method and dataset, we see no significant differences in the timeliness of detection, suggesting that randomization testing using the empirical/asymptotic p-values does not substantially affect detection performance.

**Table 6:** Detection power with and without randomization testing, using empirical/asymptotic p-values

method	ED dataset	TH dataset	CC dataset	AF dataset
KULL MA	3.17/3.23	4.24/4.23	2.60/2.55	3.18/3.19
KULL MA-DOW	3.26/3.04	4.23/4.09	2.26/2.26	2.83/2.80
KULL MA-WK	3.21/3.23	4.02/4.23	2.58/2.55	3.08/3.19
KULL MA-WK-DOW	3.21/3.04	4.03/4.09	2.41/2.26	2.82/2.80
EBP MA	2.48/2.50	3.28/3.29	**3.42/**4.16	3.90/3.99
EBP MA-DOW	2.49/2.53	3.57/3.44	**3.14/**4.10	4.20/4.36
EBP MA-WK	2.67/2.50	3.64/3.40	3.08/**2.70**	4.62/**3.92**
EBP MA-WK-DOW	2.92/2.59	4.02/3.75	2.90/**2.47**	4.53/**4.00**
EBG MA	2.84/2.91	4.00/4.19	4.52/4.43	4.35/4.63
EBG MA-DOW	3.05/3.01	4.92/4.66	4.67/4.50	4.83/4.79
EBG MA-WK	2.91/2.87	4.20/4.24	3.25/3.16	4.51/4.43
EBG MA-WK-DOW	2.95/3.04	4.99/4.73	3.08/2.96	4.46/4.56

Average days to detection at 1 false positive per month for "medium-sized" outbreaks injected into each dataset, using empirically determined thresholds on p-value (computed by randomization testing, using empirical/asymptotic p-values [37]) and score (without randomization testing) respectively. If there is a significant difference between the detection times with and without randomization, the better-performing method is marked in bold.

In Table 7, we show the empirical score threshold (without randomization testing) and the empirical p-value threshold (using randomization testing with empirical/asymptotic p-values) needed to achieve an actual false positive rate of 1/month on each of the four datasets for each detection method. These results confirm that randomization testing is miscalibrated for the three OTC datasets, and that a nominal false positive rate much lower than *α *= .05 is needed to achieve an acceptable level of false positives in practice. While the values in the table provide some preliminary guidance for calibrating detection methods with or without randomization testing, we note that the thresholds can differ dramatically between datasets. Thus we recommend obtaining at least one full year of historical data from a given data source when possible, since this approach would account for holidays and seasonal trends, and using this historical data for calibration. In this case, randomization testing is unnecessary since using the empirically determined score threshold achieves similar detection power and is much less computationally expensive. When little or no historical data can be obtained, and sufficient computational resources are available, it might be easier to estimate an appropriate p-value threshold *α *(e.g. using *α *= 0.05 for datasets where we expect the scan statistic to be correctly calibrated) and perform randomization testing using empirical/asymptotic p-values.

**Table 7:** Score and p-value thresholds corresponding to one false positive per month

method	ED dataset	TH dataset	CC dataset	AF dataset
KULL MA	7.3/0.029	10.6/3.7 × 10^-3^	25.0/2.4 × 10^-7^	18.6/9.0 × 10^-6^
KULL MA-DOW	6.0/0.034	8.7/4.6 × 10^-3^	15.4/4.8 × 10^-5^	12.0/5.9 × 10^-4^
KULL MA-WK	7.3/0.025	10.6/5.3 × 10^-3^	25.0/2.0 × 10^-7^	18.6/1.0 × 10^-5^
KULL MA-WK-DOW	6.0/0.033	8.7/5.6 × 10^-3^	15.4/6.0 × 10^-5^	12.0/3.2 × 10^-4^
EBP MA	6.7/0.025	10.3/2.6 × 10^-3^	68.6/3.7 × 10^-13^	31.4/1.3 × 10^-11^
EBP MA-DOW	6.0/0.029	9.2/1.9 × 10^-3^	57.8/2.6 × 10^-11^	30.9/1.7 × 10^-9^
EBP MA-WK	6.4/0.030	10.2/2.8 × 10^-3^	34.9/1.4 × 10^-12^	33.9/3.0 × 10^-14^
EBP MA-WK-DOW	6.0/0.019	9.1/3.2 × 10^-3^	26.8/5.3 × 10^-11^	29.4/6.3 × 10^-10^
EBG MA	13.9/4.5 × 10^-5^	20.9/2.1 × 10^-7^	28.6/8.2 × 10^-11^	19.9/6.1 × 10^-7^
EBG MA-DOW	15.9/2.7 × 10^-6^	27.9/3.4 × 10^-11^	30.8/3.5 × 10^-13^	23.7/2.1 × 10^-8^
EBG MA-WK	13.5/6.1 × 10^-5^	21.0/3.1 × 10^-7^	16.7/1.2 × 10^-6^	17.5/2.1 × 10^-6^
EBG MA-WK-DOW	16.0/2.2 × 10^-6^	26.8/1.6 × 10^-11^	19.4/7.1 × 10^-8^	20.9/6.0 × 10^-8^

Score threshold (computed without randomization testing) and p-value threshold (computed by randomization testing, using empirical/asymptotic p-values) corresponding to an observed false positive rate of 0.0329, i.e. one false positive per month.

## Discussion

A number of other evaluation studies have compared the performance of spatial detection methods. These include studies comparing the spatial scan statistic to other spatial detection methods [38,39], comparing different sets of search regions for the spatial scan [20,36], comparing spatio-temporal and purely temporal scan statistics [40], and comparing different likelihood ratio statistics within the spatial scan framework [27,41]. To our knowledge, none of these studies compare a large number of spatial scan variants across multiple datasets and specifically examine the effects of dataset characteristics (e.g. average daily count, seasonal and day-of-week trends) and outbreak size (e.g. number of affected zip codes) on the relative performance of methods, as in the present work.

Nevertheless, it is important to acknowledge several limitations of the current study, which limit the generality of the conclusions that can be drawn from these experiments. First, this paper focuses specifically on the scenario of monitoring syndromic data from a small area (a single county) on a daily basis, with the goal of rapidly detecting emerging outbreaks of disease. In this case, we wish to detect higher than expected recent counts of health-related behaviors (hospital visits and medication sales) which might be indicative of an outbreak, whether these increases occur in a single zip code, a cluster of zip codes, or even the entire monitored county. This is different than the original use of spatial scan statistics for analysis of spatial patterns of chronic illnesses such as cancer, where we may not compare observed and expected counts, but instead attempt to detect clusters with higher disease rates inside than outside. Similarly, while we focused on county-level surveillance, responsibility for outbreak detection ranges across much broader levels of geography (e.g. state, national, and international), and larger-scale disease surveillance efforts might have very different operational requirements and limitations. Second, spatial syndromic surveillance approaches (including all of the methods considered in this study) might not be appropriate for all types of disease outbreaks. Our simulations focused on outbreaks for which these approaches are likely to have high practical utility. Such outbreaks would affect a large number of individuals (thus creating detectable increases in the counts being monitored), exhibit spatial clustering of cases (since otherwise spatial approaches might be ineffective), and have non-specific early-stage symptoms (since otherwise earlier detection might be achieved by discovering a small number of highly indicative disease findings). Third, our retrospective analysis did not account for various sources of delay (including lags in data entry, collection, aggregation, analysis, and reporting) which might be present in prospective systems. Any of these sources might result in additional delays between the first cases generated by an outbreak and its detection by a deployed surveillance system. Similarly, the absolute results (number of days to detect) are highly dependent on the number and spatial distribution of injected cases; for these reasons, the comparative performance results reported here should not be interpreted as an absolute operational metric. Fourth, while differences in the relative performance of methods between datasets demonstrate the importance of using multiple datasets for evaluation, this study was limited by data availability to consider only four datasets from a single county, three of which were different categories of OTC sales from the same year. Expansion of the evaluation to a larger number of datasets, with a higher degree of independence between datasets, would provide an even more complete picture of the relative performance of methods. Finally, this analysis used existing health datasets which were aggregated to the zip code level prior to being made available for this study. Data aggregation was necessary to protect patient privacy and preserve data confidentiality, but can result in various undesirable effects related to the "modifiable areal unit problem" (MAUP) [42], including reduced variability between areas, and decreased power to detect very small affected regions, at higher levels of aggregation. However, the likelihood ratio statistics presented here, and the various methods of computing expected counts, involve only means and variances, which are resistant to aggregation effects [43]. Additionally, Gregorio et al. [44] did not find significant effects of aggregation for spatial scan statistics when comparing zip code, census tract, and finer resolutions. Thus we believe that the comparative results presented here (if not necessarily the absolute results) will be relatively stable across different levels of aggregation.

Next, we consider several issues regarding the detection of large outbreaks affecting most or all of the monitored zip codes. In the original spatial scan setting, where the explicitly stated goal was to detect significant differences in disease rate inside and outside a region, such widespread increases might not be considered relevant, or might be interpreted as a decreased disease rate outside the region rather than an increased rate inside the region. However, our present work focuses on the detection of emerging outbreaks which result in increased counts, and when we are monitoring a small area (e.g. a single county), many types of illness might affect a large portion of the monitored area. In this case, it is essential to detect such widespread patterns of disease, and to distinguish whether differences in risk are due to higher than expected risk inside the region or lower than expected risk outside the region. Kulldorff's description of the SaTScan software [1] does include the caveat that KULL is not intended for detection of large outbreaks affecting more than 50% of the monitored population. Nevertheless, SaTScan is often used as a tool (and in some cases, as the only automated surveillance tool) for outbreak detection at the county level, and it is important for practitioners to be aware that this tool has very low power for outbreaks affecting a large proportion of the monitored area. Use of the expectation-based Poisson scan statistic instead of Kulldorff's original statistic would solve this problem and provide high detection power across the entire range of possible outbreak sizes. Finally, it has been suggested that such large outbreaks might be detected better by a purely temporal alerting method instead of a spatial or spatio-temporal method. While this is likely to be true for outbreaks affecting all or nearly all of the monitored zip codes, temporal alerting methods have much lower power for small outbreak sizes, and are unable to accurately determine which subset of the monitored area has been affected by an outbreak. While simultaneous use of spatial scan and temporal alerting methods is a practical possibility, it is important to note that this creates a multiple testing issue, and each method must operate at a lower sensitivity level to maintain a combined false positive rate of *a*. Evaluation of such combinations of multiple detection methods are beyond the scope of the present work, but we note that no prior work has demonstrated that these would be more effective than a single spatial scan method (such as EBP) with high power to detect and pinpoint both small and large affected regions, and the use of a single tool instead of multiple tools has significant practical advantages as well.

It is also informative to consider our empirical results (in which EBP outperformed KULL for large outbreak sizes on all four datasets, and for small outbreak sizes on two of four datasets) in light of the theoretical results of Kulldorff [11], who proves that KULL is an individually most powerful test for detecting spatially localized clusters of increased risk (*q*_*in *_> *q*_*out*_) as compared to the null hypothesis of spatially uniform risk (*q*_*in *_= *q*_*out *_= *q*_*all*_). While KULL is optimal for differentiating between these two hypotheses, it is not necessarily optimal for differentiating between outbreak and non-outbreak days which do not correspond to these specific hypotheses. Even when no outbreaks are occurring, the real-world health datasets being monitored are unlikely to correspond to the hypothesis of independent Poisson-distributed counts and spatially uniform risk; they may be overdispersed, exhibit spatial and temporal correlations, and contain outliers or other patterns due to non-outbreak events. Similarly, real-world outbreaks may not result in a constant, multiplicative increase in expected counts for the affected region, as assumed by KULL. Finally, we note that Kulldorff's notion of an "individually most powerful" test is somewhat different than that of a "uniformly most powerful" test, being geared mainly toward correct identification of the affected cluster as opposed to determination of whether or not the monitored area contains any clusters. Our empirical results demonstrate that high detection power in the theoretical setting (assuming ideal data generated according to known models) may not correspond to high detection power in real-world scenarios when the given model assumptions are violated.

## Conclusion

This study compared the performance of twelve variants of the spatial scan statistic on the detection of simulated outbreaks injected into four different real-world public health datasets. We discovered that the relative performance of methods differs substantially depending on the size of the injected outbreak and various characteristics of the dataset (average daily count, and whether day-of-week and seasonal trends are present). Our results demonstrate that the traditional (Kulldorff) spatial scan statistic approach performs poorly for detecting large outbreaks that affect more than two-thirds of the monitored zip codes. However, the recently proposed expectation-based Poisson (EBP) and expectation-based Gaussian (EBG) statistics achieved high detection performance across all outbreak sizes, with EBP consistently outperforming EBG. For small outbreaks, EBP outperformed Kulldorff's statistic on the two datasets with low average daily counts (respiratory ED visits and OTC thermometer sales), while Kulldorff's statistic outperformed EBP on the two datasets with high average counts (OTC cough/cold and anti-fever medication sales). Using a simple adjustment for seasonal trends dramatically improved the performance of all methods when monitoring cough/cold medication sales, and adjusting for day-of-week improved the performance of Kulldorff's statistic on the cough/cold and anti-fever datasets. In all other cases, a simple 28-day moving average was sufficient to predict the expected counts in each zip code for each day. Finally, our results demonstrate that randomization testing is not necessary for spatial scan methods, when performing small-area syndromic surveillance to detect emerging outbreaks of disease. No significant performance gains were obtained from randomization on our datasets, and in many cases the resulting p-values were miscalibrated, leading to high false positive rates and reduced detection power.

These results suggest the following practical recommendations regarding the use of spatial scan methods for outbreak detection:

1. When evaluating the relative performance of different spatial scan methods, we recommend using a variety of different datasets and outbreak characteristics for evaluation, since focusing only on a single outbreak scenario may give a misleading picture of which methods perform best.

2. The traditional (Kulldorff) spatial scan statistic has very poor performance for large outbreak sizes, and thus we recommend the use of the expectation-based Poisson (EBP) statistic instead when large outbreaks are of potential interest. If only small outbreaks are of interest, we recommend the use of EBP on datasets with low average daily counts and Kulldorff's statistic on datasets with high average daily counts.

3. Adjustments for seasonal and day-of-week trends can significantly improve performance in datasets where these trends are present.

4. If a sufficient amount of historical data is available, we recommend empirical calibration of likelihood ratio scores (using the historical distribution of maximum region scores) instead of the current practice of statistical significance testing by randomization. If little historical data is available, we recommend the use of empirical/asymptotic p-values, and a threshold much lower than *α *= .05 may be necessary to avoid high false positive rates.

We are in the process of using the evaluation framework given here to compare a wide variety of other spatial biosurveillance methods, including Bayesian [3,4,25] and nonparametric [29] scan statistics. Also, all of the methods discussed here can be extended to the space-time scan statistic setting, allowing the temporal duration of detected clusters to vary. Our evaluation framework can be used to compare these space-time cluster detection methods, but the set of injected outbreaks must also be varied with respect to their temporal characteristics such as duration and rate of growth. Based on the preliminary results in [21], we expect that longer temporal window sizes will be appropriate for outbreaks that emerge more slowly, and that small but significant gains in detection power can be achieved by considering "emerging cluster" extensions of EBP that model the increase in disease rate over time. Finally, though we have focused here on the question of which statistical methods are most effective for spatial scan assuming a fixed set of search regions, this evaluation framework can also be applied to address the orthogonal question of which set of search regions to choose. A systematic comparison using the methodology presented here, but using a much wider variety of outbreak shapes, may likewise give a better idea of what sets of regions are most appropriate for different combinations of dataset and outbreak type.

## Competing interests

The author declares that they have no competing interests.
